# Readiness for antimicrobial resistance (AMR) surveillance in Pakistan; a model for laboratory strengthening

**DOI:** 10.1186/s13756-017-0260-6

**Published:** 2017-09-29

**Authors:** Dania Khalid Saeed, Rumina Hasan, Mahwish Naim, Afia Zafar, Erum Khan, Kausar Jabeen, Seema Irfan, Imran Ahmed, Mohammad Zeeshan, Zabin Wajidali, Joveria Farooqi, Sadia Shakoor, Abdul Chagla, Jason Rao

**Affiliations:** 1 Department of Pathology and Laboratory Medicine, Aga Khan University Hospital, Stadium Road, PO Box 3500, Karachi, 74800 Pakistan; 2 Baqai Institute of Health Sciences, Baqai Medical University, Karachi, Pakistan; 3 Health Security Partners, Washington, DC, 20009 USA

**Keywords:** Antimicrobial susceptibility testing, Laboratory capacity, Surveillance for antimicrobial resistance

## Abstract

**Background:**

Limited capacity of laboratories for antimicrobial susceptibility testing (AST) presents a critical diagnostic bottleneck in resource limited countries. This paper aims to identify such gaps and to explore whether laboratory networks could contribute towards improving AST in low resource settings.

**Methods:**

A self-assessment tool to assess antimicrobial susceptibility testing capacity was administered as a pre-workshop activity to participants from 30 microbiology laboratories in 3 cities in Pakistan. Data from public and private laboratories was analyzed and capacity of each scored in percentage terms. Laboratories from Karachi were invited to join a support network. A cohort of five laboratories that consented were provided additional training and updates sessions over a period of 15 months. Impact of training activities in these laboratories was evaluated using a point scoring (0-11) tool.

**Results:**

Results of self-assessment component identified a number of areas that required strengthening (scores of ≤60%). These included; readiness for AMR surveillance; 38 and 46%, quality assurance; 49 and 55%, and detection of specific organisms; 56 and 60% for public and private laboratories respectively. No significant difference was detected in AST capacity between public and private laboratories [ANOVA; *p* > 0.05]. Scoring tool used to assess impact of training within the longitudinal cohort showed an increase from a baseline of 1-5.5 (August 2015) to improved post training scores of 7-11 (October 2016) for the 5 laboratories included. Moreover, statistical analysis using paired t-Test Analysis, assuming unequal variance, indicated that the increase in scored noted represents a statistically significant improvement in the components evaluated [*p* < 0.05].

**Conclusion:**

Strengthening of laboratory capacity for AMR surveillance is important. Our data shows that close mentoring and support can help enhance capacity for antimicrobial sensitivity testing in resource limited settings. Our study further presents a model wherein laboratory networks can be successfully established and used towards improving diagnostic capacity in such settings.

**Electronic supplementary material:**

The online version of this article (10.1186/s13756-017-0260-6) contains supplementary material, which is available to authorized users.

## Background

A multi-sectorial problem, encompassing both environment and health related activities, successful containment of antimicrobial resistance (AMR) requires informed horizontal as well as vertical interventions [1], underpinned by an effective surveillance system [2, 3]. Countries that have managed to control or curtail resistance rates are those that have been able to implement a functional surveillance system; there by enabling them to gather reliable and accurate data on resistance trends as well as to monitor temporal shifts in resistance and implement strategies accordingly.

While surveillance includes several components; susceptibility reporting, data management and analysis [4], standardized and reliable laboratory practices is integral to establishing AMR surveillance, as exemplified by Sweden where the successful establishment of surveillance programs was underscored by the existence of operational and well-equipped microbiological diagnostic laboratories [5]. On the other hand, in resource limited countries (RLCs) a dearth of standardized microbiology laboratories hampers the establishment of such a system.

The significance of the microbiology diagnostic laboratory is not limited to surveillance, but also extends to other AMR curtailing strategies including antimicrobial stewardship and infection control practices. Denning et al. [6] present a very strong case for improving diagnostic capacity of laboratories by elucidating the relationship between misdiagnosis and inappropriate use of antibacterials and antifungals arising from a lack of fungal diagnostic capacity in laboratories. Subsequently, upending efforts made by antibacterial stewardships programs to control resistance. Poor communication between physician and clinical microbiologists in a centralized laboratory system, was acknowledged to undermine infection control efforts [7]. Additionally, poor access to quality assured susceptibility testing resulting in a preference for emperical and combination therapies further contributes towards increasing resistance within the population. Therefore, capacity building of laboratories can also further the case for implementing antimicrobial conservation practices in low and middle income countries (LMICs). Currently, Pakistan is in the process of implementing Global Antimicrobial Surveillance System (GLASS) [8]. Pakistan’s AMR capabilities have been identified to require urgent attention with regards to strengthening infrastructure of diagnostic laboratories in public health and animal health sector [9].

A number of publications [10–12] have discussed AMR capacity building in resource limited settings (RLS) and addressed strengthening laboratories’ capacity as part of the objectives for establishing proper surveillance systems in these countries. While, studies have been carried out that explore laboratory capacity to diagnose specific organisms [13] for control of neglected tropical diseases [14], there is a dearth of literature investigating existing gaps in AST proficiency testing in RLCs. Such gaps hinder implementation of an effective and sustainable surveillance system. The study presented evaluates laboratory capacity and gaps in both public and private sector laboratories in Pakistan. Knowledge-based interventions were introduced in selected cohort laboratories from Karachi along with periodic on-site visits of the laboratories to evaluate impact of these interventions.

## Methods

Capacity for antimicrobial susceptibility (AST) in Karachi and gaps were evaluated and identified by collecting and reviewing previous data and reports on this topic. A questionnaire based on the *SLIPTA* checklist [15], *WHO AMR surveillance: questionnaire for assessment of national network* [16], and *WHO guide for establishing laboratory-based surveillance for antimicrobial resistance* [12] was then developed. The questionnaire (Additional file 1) included the following 9 components for evaluating laboratory capacity; use of standardized methods, standardized operating procedures (SOPs), quality assurance, readiness for AMR surveillance, testing for specific organisms, equipment maintenance, technical capacity of staff, infrastructure and laboratory biosafety. This questionnaire was administered as part of a pre-workshop self-assessment questionnaire in three major cities of Pakistan; Karachi (March, 2015), Lahore (September, 2016) and Peshawar (August 2016). Participants from 30 laboratories participated in this exercise (Table 1). Scoring of responses was as follows; Yes: 1, Partial: 0.5, and No: 0.

**Table 1:** Laboratories participating in self-assessment exercise (*n* = 30)

Laboratories	Karachi		Peshawar		Lahore	
	Private	Public	Private	Public	Private	Public
Tertiary care/teaching hospital	2	5	4	2	1	8
General hospital	2	1				
Commercial diagnostic laboratories	1		1		1	
Research laboratories		1		1		
Total	5	7	5	3	2	8

Percentage scores were then calculated for each question and categories. The percentage score for each category was calculated as the sum of components scores obtained for the category expressed as a percentage of the maximum score expected for that category based on number of responses (Additional file 2). Where there were more than one participants from a particular laboratory, and the scores for individual components were different, the lower score was entered for that question. This was on the assumption that discrepancy highlights deficiency in practices and thus a gap. Interpretation of the results was as follows; response < 50%: significant improvement required, > 50 to < 80%: some improvement required, response > 80%: laboratory is in good standing.

The laboratories from Karachi were subsequently invited to participate in a 2 years follow-up project wherein further training and guidance was offered through additional workshops and on-site visits. Based on informed consent cohort of five laboratories from Karachi were identified; 4/5 laboratories were from the public sector; (3 tertiary-care hospital and one research laboratory). The remaining fifth laboratory was from the private sector. Subsequently, a bi-pronged approach involving knowledge strengthening and follow-up on-site visits were carried out on regular basis as part of capacity strengthening strategy. Activities organized for strengthening laboratory capacity in this cohort are enlisted in Table 2. In addition the staff of the cohort laboratories were encouraged to visit or make phone contact if clarifications or help with problem solving was required. To assess impact of the knowledge based interventions, on-site laboratories assessment was carried out at 3 time points during the follow-up period. An evaluation tool (Additional file 3) was used. Responses were scored as follows; Yes: 1, Partial: 0.5 and No: 0. Total score of each laboratory was calculated and used as a measure of impact over time.

**Table 2:** Knowledge based capacity strengthening activities for cohort laboratories

DATE	ACTIVITIES	
July 2015	Groups meeting with cohort laboratories that had earlier submitted their consent form to discuss project details and activity plans.	
August 2015	Cohort laboratories visited for base-line needs assessment. Evaluation tool administered	
August 2015	Gaps and solutions to address deficiencies discussed with cohort laboratories. Topics for training sessions finalized	
September- November 2015	Training sessions	Preparation of SOPs
		Using on-line resources including ASM resources
		Principles of AST’s
		Update on McFarland SOPs
	Practical session	Preparation of McFarland Standards
	Training sessions	Quality Control of AST media
		Storage of QC strains
	Workshop	Minimum Inhibitory Concentration (MIC) for Antimicrobial Sensitivity Testing
March 2016	Laboratory strengthening workshop	Antimicrobial Resistance (AMR) Updates
June 2016	Post-workshop audit of cohort laboratories conducted and evaluation tool administered. Gaps noted and laboratory workers counseled accordingly.	
October 2016	Follow up of cohort laboratories conducted to evaluate improvements and persisting gaps using evaluation tool. Laboratory workers counseled accordingly.	
December 2016	Workshops held in parallel;	Infection control Antimicrobial susceptibility testing of Fastidious organisms

### Data analysis

Statistical difference between public and private laboratories’ capacity for AST was determined by one-way ANOVA of the percentage scores for the assessed categories.

Aggregated scores from post workshop evaluations of the Karachi laboratories at time points 1 and 3 were analyzed using paired t-Test assuming unequal variances, to determine whether the impact of knowledge based interventions coupled with on-site visits was statistically significant.

## Results

**Fig. 1:**
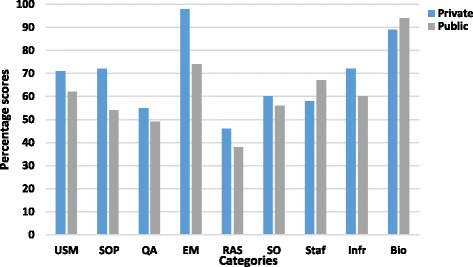
Self-assessment scores for private and public laboratories for the categories used to evaluate AST capacity. Key; USM = Use of standardized methods, SOP = Standard Operating Procedure, QA = Quality Assurance, EM = Equipment Maintenance, RAS = Readiness for AMR surveillance, SO = Specific organisms, Staf = Staffing, Infr = Infrastructure, Bio = Biosafety

Results of the pre-workshop self-assessment questionnaire show the gaps documented in the nine categories evaluated Fig. 1 (Additional file 2). Performance of both public and private laboratories in the different categories assessed was surprisingly similar. One way ANOVA [*p* = 0.953] revealed that no significant disparity existed in the capacity between public and private labs to carry out standardized AST. The majority of areas assessed scored between 50 and 80% (i.e. some improvement required). Both public and private sector laboratories scored well in biosafety areas assessed i.e. hand hygiene and incineration. However, for incineration/autoclaving prior to waste disposal; private sector laboratories scored 58% while public sector labs scored 79% (Additional file 2). Performance in both private and public sector laboratories was < 60% for quality assurance, and detection of specific organisms. Infrastructural standing of public labs (60%) was comparatively lower than private labs (72%), with unavailability of automated systems as a significant gap in both public (0%) and private labs (25%). While a score of under 50% (i.e. significant improvement required) was achieved for readiness for AMR surveillance, few laboratories generated routine antibiograms; 38 and 53% score for private and public laboratories respectively (Additional file 2). Specific gaps identified in terms of quality assurance included lack of quality control of susceptibility discs and antimicrobial disc potency, non-availability of standard guidelines in the procedure manuals for inconsistent AST results, and the need for participation in internal and external quality assurance programs (Additional file 2).

Amongst the laboratory cohort from Karachi, the initial on-site visits conducted after the workshop, revealed that the impact of the workshop was limited and that the participant laboratories only partially succeeded in implementing practices communicated during the workshops. Contributory factors included lack of resources e.g. access to standardized quality control strains for AST. Implementation of quality controls for media and antimicrobial sensitivity testing were also incomplete. The laboratories were not participating in external quality control program. Furthermore waste was not being safely disposed. In view of these observations, additional workshops, training and discussion sessions were organized for this group (Table 2).

**Fig. 2:**
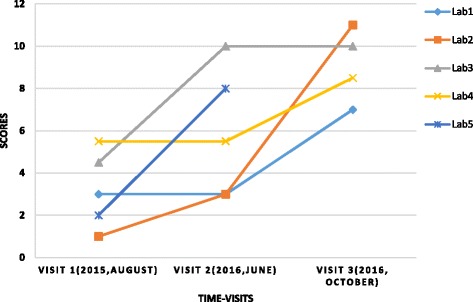
Score achieved by the cohort laboratories (1-5) at three time points. Lab 5 could only be visited at 2 time points

During the course of the study period, encouraging progress was recorded in the cohort laboratories from baseline score of ranges of 1-5.5 in August 2015 to 7-11 in October 2016 (Fig. 2). Paired t-Test, assuming unequal variance, of laboratory evaluation scores at time points 1 and 3 indicated statistically significant progress [*p* = 0.00518, t Stat = −4.2841] for four of the five cohort labs (the 5th lab was only visited at 2 time points). Changes implemented in these laboratories included; development and implementations of SOPs, quality assurance for AST, compliance with waste disposal protocols, and greater use of on-line materials including American Society of Microbiology (ASM) resources. Two laboratories furthermore started incorporating susceptibility data into an antibiogram. During this period 2 of the laboratories also began to participate in a national external quality assurance program.

## Discussion

Pakistan has a mixed public-private health care system. The public health sector comprises of a three tiered structure; primary (basic health units), secondary (tehsil and district hospitals) and tertiary (tertiary hospitals) care [17]. Due to limited spending on health by the government [18] monetary and personnel resources required to establish and maintain standardized laboratory practices are insufficient. Furthermore, with 86.8% [19] of health related expenses borne out of pocket, many resort to health services provided by the private sector. Laboratories from both public and private sector included in our study scored either < 50% (requiring significant improvement) or between 50-80% (needing some improvement) in 7 of the 9 categories evaluated. In particular limited use of quality control (QC) strains and standardized inoculum are pertinent gaps that undermine the reliability and reproducibility of AST being carried out. Additionally uninterrupted power supply in the public sector laboratories is a significant gap to be circumvented for improving laboratory infrastructure. These findings are consistent with earlier reports [20–22]. Low participation in AMR surveillance, a weak collaborative network between laboratories and insufficient use of Laboratory Information Systems (LIS) are underlying bottlenecks that need to be addressed in order to strengthen data collection, validation and aggregation of regional and national resistance data. LIS in particular is recognized to not only improve capacity for AMR surveillance through the collation of data from different laboratories but to also contribute towards standardization and improvement of the quality control of methodology [23].

While an earlier knowledge and practices (KAP) survey from Pakistan reports considerable gaps between awareness and implementation of standardized laboratory practices [24] comparatively higher scores of 54 and 72% for public and private laboratories, respectively, indicated in our study, suggest that implementation of SOPs has improved since the earlier KAP survey.

In resource limited healthcare settings where national accrediting bodies exist, a higher percentage of accredited laboratories have been observed to belong to the private sector [25]. In such settings, private sector laboratories, have the potential to assume a pivotal role in combating AMR by partnering with public sector laboratories and by participating in regional or national surveillance to produce a clearer picture of resistance trends [25]. In contrast, data from our study indicates that participation in external quality assurance was weak not only in public laboratories (46%) but also in the private sector labs (38%). Paradoxically, despite a high participation of public sector labs in internal quality assurance programs (92%), prevalence of significant gaps in these labs reiterates that internal quality assurance can be more robust and effective when complimented with an External Quality Assurance System (EQAS).

Knowledge based-interventions in the form of short courses addressing specific diseases, along with skill development have been proven to be effective models for laboratory capacity building [11]. Consistent with these findings, significant efforts by cohort laboratories towards addressing gaps; development and implementation of SOPs regular use of standardized quality control strains and standardized inoculum for AST emphasizes the value of knowledge-based interventions towards addressing laboratory gaps. The success of proficiency testing (PT) in conjunction with training programs has been highlighted by a number of studies from resource limited settings [26–29]. The approach of partnering laboratories fulfilling core capacity with weaker labs suggests a model of a sustainable network for knowledge and skill transfer for RLCs. It has been suggested that such partnering of laboratories may also contribute towards reducing costs and increasing the range of diagnostic facilities; enabling a robust laboratory system for surveillance of infectious diseases [30]. Furthermore, tiered laboratory networks along with clearly defined national guidelines that push for gearing lab capacity towards national accreditation can achieve remarkable improvements in laboratory diagnostic capacity for surveillance.

The initial part of our study relied on a self assessment tool to evaluate laboratory capacity. An inherent drawback of this approach is that it could have been influenced by individual bias as well as knowledge and experience of the respondents. However, the fact that the cohort laboratories were able to successfully address many of their gaps was encouraging and suggests a model wherein laboratory networks can be established and leveraged towards improving diagnostic capacity in resource limiting settings. It is important to note that the role of the cohort laboratories leadership was essential for success of this model. The laboratory leadership encouraged their staff to participate in training activities and supported implementation of changes needed to address identified gaps. Such facilitation was key in strengthening of the laboratory network.

## Conclusion

Our study illustrates that in resource constraint settings developing a laboratory network wherein laboratories that fulfill the International Health Regulation (IHR) core capacity provide a central support role, providing didactic trainings and workshops combined with long term follow-up and mentoring can successfully strengthen laboratory capacity.

## Additional files

Additional file 1: Self-administered questionnaire used to assess laboratory capacity for AMR testing. Presents the self-administered questionnaire used for evaluating laboratory capacity. (DOCX 44 kb)

Additional file 2: Scoring method used including percentage scores for each question in a given category. Total scores and percentage scores for each category of laboratory capacity for both public and private sector are also presented. Presents the scores including total percentage of public and private sector in each category as well as the individual scores in the different components that constituted a particular category. (DOCX 25 kb)

Additional file 3: Questionnaire to evaluate impact of knowledge based intervention on laboratory performance during study period. Presents questionnaire used for evaluating impact of knowledge based intervention on laboratory performance during the study period. (DOCX 12 kb)

## Abbreviations

AMR: Antimicrobial resistance
ASM: American Society of Microbiology
AST: Antimicrobial susceptibility testing
CLSI: Clinical Laboratory Standards Institute
EQAS: External quality assessment system
GDP: Gross domestic product
GLASS: Global Antimicrobial Surveillance System
IHR: International Health Regulation
KAP: Knowledge and practices
LIS: Laboratory Information System
LMICs: Low middle income countries
PBSA: Pakistan Biological Safety Association
PT: Proficiency testing
RLC: Resource limited countries
RLS: Resource limited settings
SLIPTA: Stepwise Laboratory Quality Improvement Process Towards Accreditation
SOPs: Standardized operation procedures
WHO: World Health Organization

## References

[1] Dar OA, Hasan R, Schlundt J, Harbarth S, Caleo G, Dar FK, et al. Antimicrobials: access and sustainable effectiveness. Exploring the evidence base for national and regional policy interventions to combat resistance. Lancet. 2016; 10.1016/S0140-6736(15)00520-6 10.1016/S0140-6736(15)00520-6 26603921

[2] WHO Global strategy for containment of antimicrobial resistance. Geneva: World Health Organization; 2001. http://www.who.int/drugresistance/WHO_Global_Strategy_English.pdf. Accessed 7 May 2017.

[3] Mcgowan JE, Tenover FC. Confronting bacterial resistance in healthcare settings: a crucial role for microbiologists. Nat Rev Microbiol. 2004; doi: 10.1038/nrmicro845. 10.1038/nrmicro845 15083160

[4] Johnson A. Surveillance of antibiotic resistance. Philos Trans R Soc Lond Ser B Biol Sci. 2015; 10.1098/rstb.2014.0080. [Abstract]. 10.1098/rstb.2014.0080 PMC4424431 25918439

[5] Struwe J. Fighting antibiotic resistance in Sweden – past, present and future. J Wien Klin Wochenschr. 2008; doi.org/10.1007/s00508-008-0977-6 10.1007/s00508-008-0977-6 18545950

[6] Denning DW, Perlin DS, Muldoon EG, Colombo AL, Chakrabarti A, Richardson MD, et al. Delivering on antimicrobial resistance agenda not possible without improving fungal diagnostic capabilities. Emerg Infect Dis. 2017; 10.3201/eid2302.152042 10.3201/eid2302.152042 PMC5324810 27997332

[7] Peterson LR, Hamilton JD, Baron EJ, Tompkins LS, Miller JM, Wilfert CM, et al. Role of clinical microbiology laboratories in the management and control of infectious diseases and the delivery of health care. Clin Infect Dis. 2001; 10.1086/318725 10.1086/318725 11181125

[8] World Health Organization (2015). Global antimicrobial resistance surveillance system manual for early implementation.

[9] Joint external evaluation of IHR core capacities of the Islamic Republic of Pakistan. Mission report. World Health Organization; 2016. http://www.who.int/ihr/publications/WHO-WHE-CPI-2017.9/en/. Accessed 7 May 2017.

[10] London School of Hygiene and Tropical Medicine. AMR Surveillance in low- and middle income settings*.* A roadmap for participation in the Global Antimicrobial Surveillance System (GLASS). Flemming Fund. 2016; http://amr.lshtm.ac.uk/2016/11/17/report-amr-surveillance-low-middle-income-countries/. Accessed 7 May 2017. 10.12688/wellcomeopenres.12527.1 PMC5645727 29062918

[11] Dacombe R, Bates I, Bhardwaj M, Wallis S, Pulford J (2016). An analysis of approaches to laboratory capacity strengthening for drug resistant infections in low and middle income countries.

[12] Guide for establishing laboratory-based surveillance for antimicrobial resistance. Africa: World Health Organization; 2013. http://apps.who.int/medicinedocs/documents/s20135en/s20135en.pdf. Accessed 7 May 2017.

[13] Slotved H-C, Yatich KK, Sam SO, Ndhine EO. The capacity of diagnostic laboratories in Kenya for detecting infectious diseases. Trop Med Health. 2017; doi.org.10.1186/s41182-017-0049-6 10.1186/s41182-017-0049-6 PMC5410037 28461779

[14] Njelesani J, Dacombe R, Palmer T, Smith H, Koudou B, Bockarie M, et al. A systematic approach to capacity strengthening of laboratory systems for control of neglected tropical diseases in Ghana, Kenya, Malawi and Sri Lanka. PLoS Negl Trop Dis. 2014; doi: 10.1371/journal.pntd.0002736.t002. 10.1371/journal.pntd.0002736 PMC3945753 24603407

[15] Stepwise Laboratory Quality Improvement Process Towards Accreditation (SLIPTA) checklist for clinical and public health laboratories. Version 2, p.1-48. Africa: World Health Organization; 2015. http://www.afro.who.int/index.php?option=com_docman&task=doc_download&gid=9778&Itemid=2593. Accessed 7 May 2017.

[16] Antimicrobial Resistance Surveillance (2003). Questionnaire for assessment of national networks. Global health security.

[17] Nishtar S (2006). The gateway paper; health system in Pakistan – a way forward. Pakistan’s health policy forum and Heartfile.

[18] Health expenditure, total (% of GDP). World Bank; 2014. http://data.worldbank.org/indicator/SH.XPD.TOTL.ZS. Accessed 7 May 2017

[19] Out of pocket health expenditure (% of private expenditure on health). World Bank; 2014. http://data.worldbank.org/indicator/SH.XPD.OOPC.ZS. Accessed 7 May 2017.

[20] Ndihokubwayo JB, Yahaya AA, Dester AT, Ki-Zerbo G, Asamoah-Odei E, Keita B (2013). Antimicrobial resistance in the African region: issues, challenges and actions proposed. Afr Health Monit.

[21] Solomon S, Ijaz K, Carlet J, Upham G (2015). Surveillance and monitoring of antimicrobial resistance (AMR). AMR Control 2015, Overcoming Global Antimicrobial Resistance.

[22] Okeke IN. Laboratory system as an antimicrobial containment tool in Africa. Afr J Lab Med. 2016; 10.4102/ajlm.v5i3.497 10.4102/ajlm.v5i3.497 PMC5433813 28879140

[23] Nyasulu PS, Paszko C, Mbelle N. A narrative review of the laboratory information system and its role in antimicrobial resistance surveillance in South Africa. Adv Microbiol. 2014; 10.4236/aim.2014.410074

[24] Ghanchi NK, Khan E, Farooqi JQ, Fasih N, Dojki M, Hughes MA. Knowledge and practices of laboratory workers on standardized antimicrobial susceptibility testing and biosafety practices to prevent the spread of superbugs in Pakistan. Am J Infect Control. 2014; 10.1016/j.ajic.2014.06.006 10.1016/j.ajic.2014.06.006 25179341

[25] Gandra S, Merchant AT, Laxminarayan R. A role for private sector laboratories in public health surveillance of antimicrobial resistance. Future Microbiol. 2016; 10.2217/fmb.16.17 10.2217/fmb.16.17 27192102

[26] Chaitram JM, Jevitt LA, Lary S, Tenover FC. The World Health Organization’s external quality assurance system proficiency testing program has improved the accuracy of antimicrobial susceptibility testing and reporting among participating laboratories using NCCLS methods. J Clin Microbiol. 2003; 10.1128/JCM.41.6.2372 10.1128/JCM.41.6.2372-2377.2003 PMC156569 12791851

[27] Garcia A, Subbarao S, Zhang G, Parsons L, Nkengasong J, Ou C-Y, et al. Impact of proficiency testing program for laboratories conducting early diagnosis of HIV-1 infection in infants in low- to middle-income countries. J Clin Microbiol. 2014; doi: 10.1128/JCM.03097-13. 10.1128/JCM.03097-13 PMC3957753 24353004

[28] Stevenson KB, Samore M, Barbera J, Moore JW, Hannah E, Houck P, et al. Detection of antimicrobial resistance by small rural hospital microbiology laboratories: comparison of survey responses with current NCCLS laboratory standards. Diagn Microbiol Infect Dis. 2003; doi.org/10.1016/S0732-8893(03)00092-0 10.1016/s0732-8893(03)00092-0 12967743

[29] Masanza MM, Nqobile N, Mukanga D, Gitta SN. Laboratory capacity building for the International Health Regulations (IHR[2005]) in resource-poor countries: the experience of the African Field Epidemiology Network (AFENET). BMC Public Health. 2010; doi: 10.1186/1471-2458-10-S1-S8. 10.1186/1471-2458-10-S1-S8 PMC3005580 21143830

[30] Hsieh K, Kimsey P, Buehring G. Using Interorganizational partnerships to strengthen public health laboratory systems. Public Health Rep. 2013; doi: 10.1177/00333549131280S210. 10.1177/00333549131280S210 PMC3730007 23997305

