# Supplementary material for: Readiness for antimicrobial resistance (AMR) surveillance in Pakistan; a model for laboratory strengthening
Source: Antimicrob Resist Infect Control. 2017 Sep 29;6:101. doi: 10.1186/s13756-017-0260-6 (PMC5622515; doi:10.1186/s13756-017-0260-6)
Supplement: Supplementary file 1 — Self-administered questionnaire used to assess laboratory capacity for AMR testing. Presents the self-administered questionnaire used for evaluating laboratory capacity. (DOCX 44 kb) [file 13756_2017_260_MOESM1_ESM.docx]

**Additional file 1: Self-administered questionnaire used to assess laboratory capacity for AMR testing.**

| **USE OF STANDARDIZED METHODS** | | | |
| --- | --- | --- | --- |
| Q1 | Are criteria for selection of antimicrobial agents for testing specific organisms from different body sites clearly defined? | Yes |  |
|  |  | Partial |  |
|  |  | No |  |
| Q2 | Are interpretive criteria based on current CLSI (Clinical Laboratory Standards Institute) BSAC (British Society for  Antimicrobial Chemotherapy)/other guidelines? | Yes |  |
|  |  | Partial |  |
|  |  | No |  |
| Q3 | Are single isolates or pure cultures only used for final performance of antimicrobial susceptibility testing? | Yes |  |
|  |  | Partial |  |
|  |  | No |  |
| Q4 | Is the inoculum size standardized using a turbidity standard or other acceptable method? | Yes |  |
|  |  | Partial |  |
|  |  | No |  |
| Q5 | For disk susceptibility tests, are zone sizes of controls measured and recorded? | Yes |  |
|  |  | Partial |  |
|  |  | No |  |
| Q6 | For antimicrobial susceptibility testing of either disk or dilution type, are control organisms tested with each new lot or batch of antimicrobials or media, and each day the test is performed? | Yes |  |
|  |  | Partial |  |
|  |  | No |  |
| Q7 | Are zone sizes of tests measured and used for recording sensitivity resistance? | Yes |  |
|  |  | Partial |  |
|  |  | No |  |
| Q8 | Does your lab use 0.5 McFarland standard for AST? | Yes |  |
|  |  | Partial |  |
|  |  | No |  |
| Q9 | Does your lab use commercially prepared dehydrated AST media? | Yes |  |
|  |  | Partial |  |
|  |  | No |  |
| Q10 | Does your lab perform Susceptibility Testing directly from specimen based on clinical information? | Yes |  |
|  |  | Partial |  |
|  |  | No |  |
| Q11 | If direct susceptibility testing from specimen show mixed cultures, does your lab repeat susceptibility testing with isolated organisms? | Yes |  |
|  |  | Partial |  |
|  |  | No |  |
| **USE OF STANDARDIZED OPERATING PROCEDURES (SOPs)** | | | |
| Q12 | For antimicrobial susceptibility testing systems, are there documented criteria in your institutions Standard Operating Procedure for interpretation of the endpoint or zone size? | Yes |  |
|  |  | Partial |  |
|  |  | No |  |
| Q13 | Are guidelines established for the number and type of antibiotics reported for organisms isolated from different sites of infection? | Yes |  |
|  |  | Partial |  |
|  |  | No |  |
| Q14 | Do you report Antimicrobial Susceptibility Testing results based on Hospital policy (in consultation with Pharmacy, Infection control and Infectious diseases physicians. | Yes |  |
|  |  | Partial |  |
|  |  | No |  |
| **QUALITY ASSURANCE** | | |  |
| Q15 | Is each new lot of susceptibility disks checked for activity before use? | Yes |  |
|  |  | Partial |  |
|  |  | No |  |
| Q16 | Does your lab use QC (quality control) strains to assess new lot of susceptibility discs? | Yes |  |
|  |  | Partial |  |
|  |  | No |  |
| Q17 | Are tolerance limits for potency of antimicrobials established (criteria for "out of control")? | Yes |  |
|  |  | Partial |  |
|  |  | No |  |
| Q18 | Does the procedure manual address unusual or inconsistent antimicrobial testing results? | Yes |  |
|  |  | Partial |  |
|  |  | No |  |
| Q19 | Does your lab participate in any Antimicrobial Susceptibility Testing related internal quality assurance program? | Yes |  |
|  |  | Partial |  |
|  |  | No |  |
| Q20 | Does your lab participate in any Antimicrobial Susceptibility Testing related external quality assurance program? | Yes |  |
|  |  | Partial |  |
|  |  | No |  |
| Q21 | Are out of control results reported to supervisory personnel? Is there evidence of corrective action taken? Some examples include: 1.*Escherichia coli* that appears resistant to imipenem, 2.*Klebsiella* spp. susceptible to ampicillin 3.*Proteus mirabilis* resistant to ampicillin 4.*Staphylococcus aureus* resistant to vancomycin | Yes |  |
|  |  | Partial |  |
|  |  | No |  |
| **READINESS FOR AMR SURVEILLANCE** | | | |
| Q22 | Does your lab participate in antimicrobial resistance surveillance? | Yes |  |
|  |  | Partial |  |
|  |  | No |  |
| Q23 | Does your lab generate on routine basis antibiogram for purpose of monitoring the resistant and sensitivity patterns in your institution? | Yes |  |
|  |  | Partial |  |
|  |  | No |  |
| Q24 | Does your lab conduct all Antimicrobial Susceptibility Testing or forwards it to other labs? | Yes |  |
|  |  | Partial |  |
|  |  | No |  |
| Q25 | Does your lab receive samples for Antimicrobial Susceptibility Testing from other labs? | Yes |  |
|  |  | Partial |  |
|  |  | No |  |
| Q26 | Is Antimicrobial Susceptibility Testing cumulative data collected manually? | Yes |  |
|  |  | Partial |  |
|  |  | No |  |
| Q27 | Is Antimicrobial Susceptibility Testing cumulative data collected automatically using lab information system (LIS)? | Yes |  |
|  |  | Partial |  |
|  |  | No |  |
| **DETECTION OF SPECIFIC ORGANISMS** | | | |
| Q28 | Does your lab test for Methicillin-Resistant *Staphylococcus aureus* (MRSA)? | Yes |  |
|  |  | Partial |  |
|  |  | No |  |
| Q29 | Does your lab test for Vancomycin Resistant *Enterococcus* (VRE)? | Yes |  |
|  |  | Partial |  |
|  |  | No |  |
| Q30 | Does your lab provide testing for β lactamase in selected organisms? | Yes |  |
|  |  | Partial |  |
|  |  | No |  |
| Q31 | Does your lab provide Antimicrobial Susceptibility Testing for Extended Spectrum of β-lactamase (ESBL) producing Gram Negative Bacilli? | Yes |  |
|  |  | Partial |  |
|  |  | No |  |
| Q32 | Does your lab provide Antimicrobial Susceptibility Testing for *Klebsiella pneumonia* producing carbapenemase (KPC) | Yes |  |
|  |  | Partial |  |
|  |  | No |  |
| **EQUIPMENT MAINTENANCE** | | | |
| Q33 | Are Antimicrobial Susceptibility Testing equipment maintained appropriately and calibrated? | Yes |  |
|  |  | Partial |  |
|  |  | No |  |
| Q34 | Does your lab monitor incubator temperatures on a daily basis? | Yes |  |
|  |  | Partial |  |
|  |  | No |  |
| **STAFFING** | | | |
| Q35 | Is your technical lab staff trained for conducting Antimicrobial Susceptibility Testing? | Yes |  |
|  |  | Partial |  |
|  |  | No |  |
| Q36 | Is your staff compliment doing Antimicrobial Susceptibility Testing adequate for number of tests done in your lab? | Yes |  |
|  |  | Partial |  |
|  |  | No |  |
| **INFRASTRUCTURE** | | | |
| Q37 | Does your lab have automated system (Vitek, Microscan, Maldi-Tof, etc.) to conduct Antimicrobial Susceptibility Testing? | Yes |  |
|  |  | Partial |  |
|  |  | No |  |
| Q38 | Does your lab have refrigerators for disc storage? | Yes |  |
|  |  | Partial |  |
|  |  | No |  |
| Q39 | Does your lab have autostart backup generator for refrigerators? | Yes |  |
|  |  | Partial |  |
|  |  | No |  |
| Q40 | Does your lab have autostart backup generator for incubators? | Yes |  |
|  |  | Partial |  |
|  |  | No |  |
| Q41 | Does your lab experience delays in Antimicrobial Susceptibility Testing due to lack of reagents? | Yes |  |
|  |  | Partial |  |
|  |  | No |  |
| **BIOSAFETY** | | | |
| Q42 | Does your lab autoclave/incinerate cultures prior to discard? | Yes |  |
|  |  | Partial |  |
|  |  | No |  |
| Q43 | Do you have handwashing facility in the laboratory? | Yes |  |
|  |  | Partial |  |
|  |  | No |  |
| Q44 | Does your lab get continuous supply of running water? | Yes |  |
|  |  | Partial |  |
|  |  | No |  |
| Q45 | Does your lab have soap supply in the handwash facility? | Yes |  |
|  |  | Partial |  |
|  |  | No |  |
