# Supplementary material for: Readiness for antimicrobial resistance (AMR) surveillance in Pakistan; a model for laboratory strengthening
Source: Antimicrob Resist Infect Control. 2017 Sep 29;6:101. doi: 10.1186/s13756-017-0260-6 (PMC5622515; doi:10.1186/s13756-017-0260-6)
Supplement: Supplementary file 2 — Scoring method used including percentage scores for each question in a given category. Total scores and percentage scores for each category of laboratory capacity for both public and private sector are also presented. Presents the scores including total percentage of public and private sector in each category as well as the individual scores in the different components that constituted a particular category. (DOCX 25 kb) [file 13756_2017_260_MOESM2_ESM.docx]

**Additional file 2: Scoring method used including percentage scores for each question in a given category. Total scores and percentage scores for each category of laboratory capacity for both public and private sector are also presented.**

|  | Private | | | Public | | |
| --- | --- | --- | --- | --- | --- | --- |
|  | Total responses | Score | % score | Total responses | Score | % score |
| **USE OF STANDARDISED METHODS** |  |  |  |  |  |  |
| 1. Are Criteria for selection of antimicrobial agents for testing specific organisms from different body sites clearly defined? | 12 | 9.5 | 79 | 18 | 14.5 | 81 |
| 2. Are interpretative criteria based on current CLSI/other guidelines? | 12 | 10 | 83 | 17 | 11.5 | 68 |
| 3. Are single isolates or pure cultures only used for final AST performance | 12 | 10 | 83 | 17 | 15 | 88 |
| 4.Is the inoculum size standardized using a turbidity standard or other acceptable method | 12 | 9.5 | 79 | 17 | 12 | 71 |
| 5. For disk susceptibility tests, are zone sizes of controls measured and recorded | 12 | 10.5 | 88 | 18 | 9.5 | 53 |
| 6. For antimicrobial susceptibility testing of either disk or dilution type, are control org tested with each new lot or batch of antimicrobials or media, and each day the test is performed? | 12 | 6 | 50 | 18 | 6.5 | 36 |
| 7. Are zone sizes of tests measured and used for recording sensitivity resistance? | 12 | 9 | 75 | 18 | 15 | 83 |
| 8. Does your lab use 0.5 McFarland standard for AST? | 12 | 7 | 58 | 18 | 9 | 50 |
| 9. Does your lab use commercially prepared dehydrated AST media? | 12 | 6 | 50 | 17 | 6 | 35 |
| 10. Does your lab perform Susceptibility testing directly from specimen based on clinical information? | 12 | 8 | 67 | 18 | 8.5 | 47 |
| 11. If direct susceptibility testing from specimen show mixed cultures does your lab repeat susceptibility testing with isolated organisms? | 12 | 8.5 | 71 | 18 | 12 | 67 |
| **Total for Use of standardised methods** | **132** | **94** | **71** | **194** | **119.5** | **62** |
| **SOP (STANDARDIZED OPERATING PROCEDURES)** |  |  |  |  |  |  |
| 12. For antimicrobial susceptibility testing systems, are there documented criteria in your institutions SOPs for interpretation of the endpoint or zone size? | 12 | 10 | 83 | 18 | 11 | 61 |
| 13. Are guidelines established for the number and type of antibiotics reported for organisms isolated from different sites of infection? | 12 | 10 | 83 | 18 | 10 | 56 |
| 14. Do you report AST results based on Hospital Policy (consultation with Pharmacy + Infection Control + Infectious Diseases Physicians) | 12 | 6 | 50 | 17 | 7.5 | 44 |
| **Total for SOP** | **36** | **26** | **72** | **53** | **28.5** | **54** |
| **QUALITY ASSURANCE** |  |  |  |  |  |  |
| 15. Is each new lot of susceptibility disks for activity before use? | 12 | 9 | 75 | 18 | 9.5 | 53 |
| 16. Does your lab use QC (quality control) strains to assess new lot of susceptibility discs? | 12 | 6 | 50 | 18 | 7 | 39 |
| 17. Are tolerance limits for potency of antimicrobials discs established (criteria for "out of control")? | 12 | 7.5 | 63 | 17 | 5.5 | 32 |
| 18. Does the procedure manual address unusual or inconsistent antimicrobial testing results? | 11 | 4.5 | 41 | 14 | 6 | 43 |
| 19. Does your lab participate in any AST related internal quality assurance program? | 12 | 5.5 | 46 | 18 | 16.5 | 92 |
| 20. Does your lab participate in any AST related external quality assurance program? | 12 | 5 | 42 | 17 | 6.5 | 38 |
| 21. Are out of control results reported to supervisory personnel? Is there evidence of corrective action taken? Some examples include; 1. *E.coli* resistant to Imipenem 2.*Klebsiella* sp. susceptible to ampicillin 3. *P. mirabilis* resistant to ampicillin 4.*S.aureus* resistant to vancomycin | 12 | 8 | 67 | 18 | 8 | 44 |
| **Total for Quality Assurance** | **83** | **45.5** | **55** | **120** | **59** | **49** |
| **EQUIPMENT MAINTENANCE** |  |  |  |  |  |  |
| 22. Are AST equipment maintained appropriately and calibrated? | 12 | 11.5 | 96 | 17 | 10.5 | 62 |
| 23. Does your lab monitor incubator temperatures on a daily basis | 12 | 12 | 1 | 18 | 15.5 | 86 |
| **Total for Equipment Maintenance** | **24** | **23.5** | **98** | **35** | **26** | **74** |
| **READINESS FOR AMR SURVEILLANCE** |  |  |  |  |  |  |
| 24. Does your lab participate in antimicrobial resistance surveillance? | 12 | 4.5 | 38 | 18 | 6.5 | 36 |
| 25. Does your lab generate on routine basis antibiogram for purpose of monitoring the resistant and sensitivity patterns in your institution? | 12 | 4.5 | 38 | 18 | 9.5 | 53 |
| 26. Does your lab conduct all AST testing or forward to other labs? | 12 | 8 | 67 | 17 | 5 | 30 |
| 27. Does your lab receive samples for AST from other labs? | 12 | 5 | 42 | 17 | 4.5 | 26 |
| 28. Is AST cumulative data collected manually? | 12 | 4 | 33 | 18 | 10 | 56 |
| 29. Is AST cumulative data collected automatically using LIS | 12 | 7 | 58 | 16 | 4.5 | 28 |
| **Total for Readiness for AMR surveillance** | **72** | **33** | **46** | **104** | **40** | **38** |
| **TESTING SPECIFIC ORGANISMS** |  |  |  |  |  |  |
| 30. Does your lab test for MRSA | 12 | 10.5 | 88 | 17 | 12 | 71 |
| 31.Does your lab test for VRE | 12 | 8.5 | 71 | 17 | 10.5 | 62 |
| 32. Does your lab provide testing for B lactamase in selected organisms? | 12 | 7.5 | 63 | 18 | 10 | 56 |
| 33. Does your lab provide AST for ESBL producing Gram's Negative Bacilli? | 12 | 4.5 | 38 | 18 | 11 | 61 |
| 34. Does your lab provide AST for KPC | 12 | 5 | 42 | 18 | 6 | 33 |
| **Total for Testing specific organisms** | **60** | **36** | **60** | **88** | **49.5** | **56** |
| **STAFFING** |  |  |  |  |  |  |
| 35. Is your technical staff trained for conducting AST? | 12 | 9 | 75 | 18 | 13 | 72 |
| 36. Is your staff compliment doing AST adequate for a number of tests done in your lab? | 12 | 5 | 42 | 18 | 11 | 61 |
| **Total for Staffing** | **24** | **14** | **58** | **36** | **24** | **67** |
| **INFRASTRUCTURE** |  |  |  |  |  |  |
| 37. Does your lab have automated system (Vitek, Microscan, MALDI-TOF etc) | 12 | 3 | 25 | 17 | 0 | 0 |
| 38. Does your lab have refrigerators for disc storage? | 12 | 8.5 | 71 | 18 | 16 | 89 |
| 39. Does your lab have auto-start backup generator for refrigerators | 12 | 10.5 | 88 | 18 | 12 | 67 |
| 40. Does your lab have auto-start backup generator for incubators | 12 | 11.5 | 96 | 18 | 12 | 67 |
| 41.Your lab does not experience delays in AST due to lack of reagents | 12 | 9.5 | 21 | 16 | 12.5 | 22 |
| **Total for Infrastructure** | **60** | **43** | **71.7** | **87** | **52.5** | **60.3** |
| **BIOSAFETY** |  |  |  |  |  |  |
| 42. Does your lab autoclave/incinerate cultures prior to discard? | 12 | 7 | 58 | 14 | 11 | 79 |
| 43. Do you have handwashing facility in the laboratory | 12 | 12 | 100 | 18 | 17.5 | 97 |
| 44. Does your lab get continuous supply of running water | 12 | 12 | 100 | 18 | 18 | 100 |
| 45. Does your lab have soap supply in the hand-wash facility | 12 | 11.5 | 96 | 18 | 17.5 | 97 |
| **Total for Biosafety** | **48** | **42.5** | **89** | **68** | **64** | **94** |
