# Supplementary material for: Readiness for antimicrobial resistance (AMR) surveillance in Pakistan; a model for laboratory strengthening
Source: Antimicrob Resist Infect Control. 2017 Sep 29;6:101. doi: 10.1186/s13756-017-0260-6 (PMC5622515; doi:10.1186/s13756-017-0260-6)
Supplement: Supplementary file 3 — Questionnaire to evaluate impact of knowledge based intervention on laboratory performance during study period. Presents questionnaire used for evaluating impact of knowledge based intervention on laboratory performance during the study period. (DOCX 12 kb) [file 13756_2017_260_MOESM3_ESM.docx]

**Additional file 3: Questionnaire to evaluate impact of knowledge based intervention on laboratory performance during study period**

**Laboratory name: ___________________________________ Date: _____________________**

| 1 | Do you have SOPs according to CLSI | Yes | No |
| --- | --- | --- | --- |
|  | If yes, for which procedures: (ungraded) | | |
| 2 | Do you use ASM resources | Yes | No |
|  | If yes, for which purpose: (ungraded) | | |
| 3 | Can your lab prepare 0.5 McFarland standards? | Yes | No |
| 4 | If yes, do you use it as inoculum control | Yes | No |
| 5 | How is it maintained and stored? (Please show us);  Proper maintenance | Yes | No |
| 6 | Are antibiotic panels designed? | Yes | No |
| 7 | Are you using provided ATCC controls? | Yes | No |
|  | If yes, then for which test and how often? (ungraded) | | |
| 8 | How are ATCC controls maintained and stored? (Please show us);  Proper storage | Yes | No |
| 9 | For disk susceptibility tests, is each new lot checked for activity before use? | Yes | No |
| 10 | Are zone sizes of controls measured and recorded | Yes | No |
| 11 | Does lab autoclave/incinerate cultures prior to discard? | Yes | No |
